# Case report: Thoracic and lumbar plasma cell myeloma mimicking hemangiomas on MRI and ^18^F-FDG PET/CT

**DOI:** 10.3389/fmed.2022.967531

**Published:** 2022-08-04

**Authors:** Xianwen Hu, Wei Xiong, Shun Li, Xue Li, Jiong Cai, Pan Wang, Dandan Li

**Affiliations:** ^1^Affiliated Hospital of Zunyi Medical University, Department of Nuclear Medicine, Zunyi, China; ^2^Yinjiang Autonomous County People's Hospital, Department of Medical Imaging, Yinjiang, China; ^3^Zunyi Hospital of Traditional Chinese Medicine, Department of Obstetrics, Zunyi, China

**Keywords:** plasma cell myeloma, hemangioma, PET/CT, magnetic resonance imaging, fluoro18-labeled deoxyglucose

## Abstract

Plasma cell myeloma (PCM) is a malignant clonal disease of abnormal proliferation of plasma cells, which is the second most common hematological malignancy after leukemia. PCM often diffuses and involves the bones of the whole body, especially the spinal column, ribs, skull, pelvis, and other axial bones and flat bones. Herein, we present a 55-year-old man who came to the hospital seeking medical help for low-back pain and numbness in his lower limbs. Computed tomography (CT) was performed because the clinician suspected that the patient had a herniated disc, and the results showed that the 7^th^ thoracic vertebrae and the 3^rd^ lumbar vertebrae showed a low density of bone destruction with “honeycombing” changes. Magnetic resonance imaging (MRI) showed that the corresponding lesions presented long T1 and long T2 signals, and the lesions were significantly enhanced in contrast-enhanced T1WI sequences, and fluoro18-labeled deoxyglucose positron emission tomography/computed tomography (^18^F-FDG PET/CT) showed mild radioactive uptake in the lesions. Based on these imaging findings, the patient was considered for a diagnosis of hemangiomas, and surgery was performed because the affected vertebra was pressing on the spinal cord. However, intraoperative frozen section examination showed that the patient had plasma cell myeloma. Our case study suggests that PCM involving a single thoracic and lumbar spine is rare and should be considered as one of the imaging differential diagnoses of hemangiomas. Moreover, the diagnosis of PCM is difficult when the number of lesions is small, especially when the plasma cell ratio is within the normal reference range in laboratory tests.

## Case description

**Figure 1:**
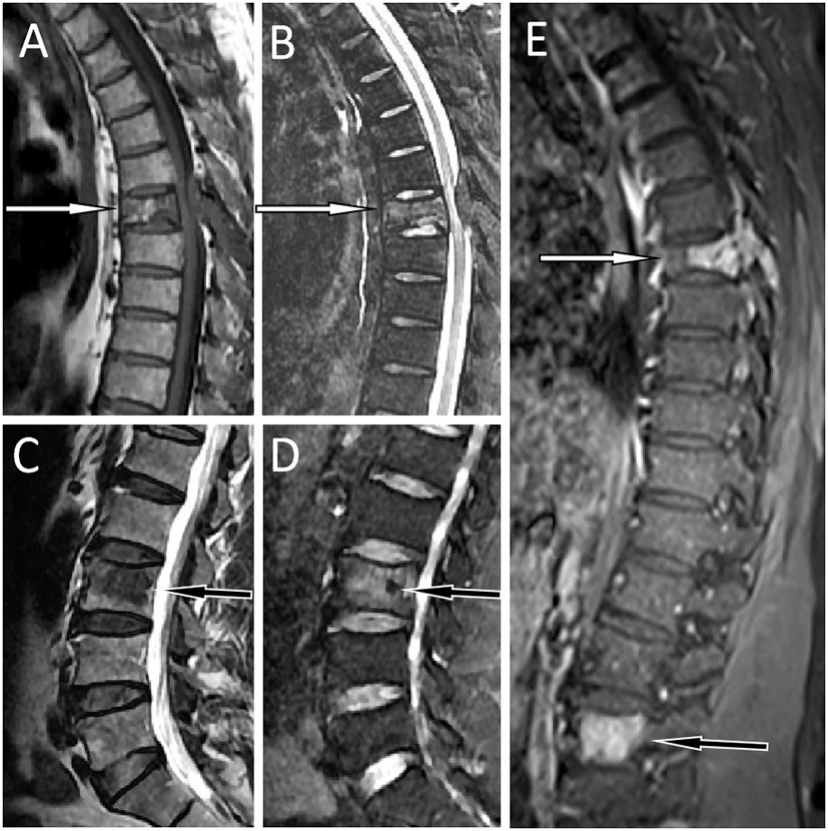
MRI showed abnormal signal shadows in the 7th thoracic (T7) and adnexa vertebrae [**(A)** T1WI; **(B)** T2WI fat suppression sequence; white arrows] and the 3rd lumbar vertebrae [**(C)** T1WI; **(D)** T2WI fat suppression sequence; black arrows]. Besides, The T7 vertebrae became wedge-shaped, suggesting a pathological compression fracture. T1WI contrast enhancement showed obvious homogeneous enhancement of the lesions **(E)**. T7, the white arrow; L3, the black arrow.

**Figure 2:**
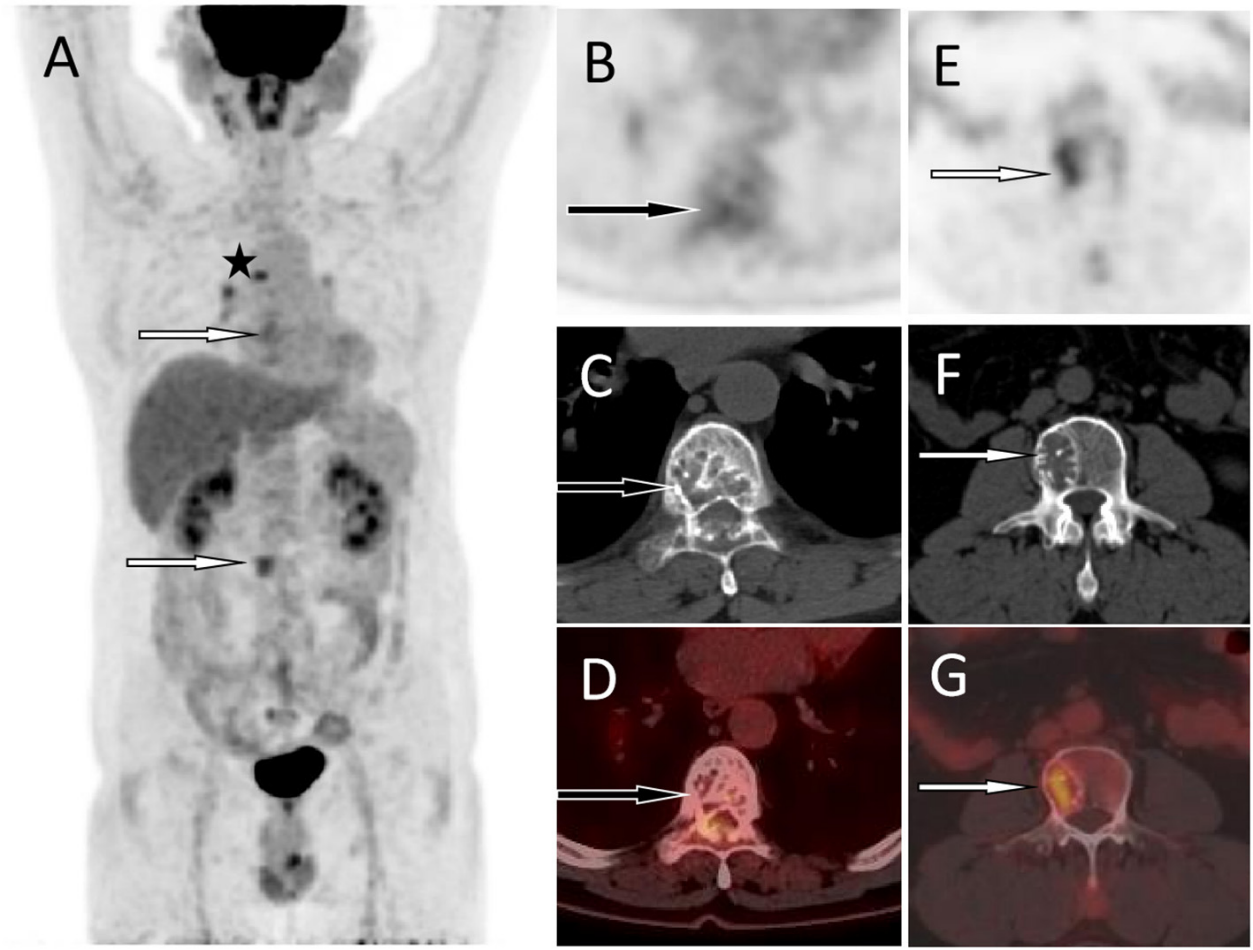
^18^F-FDG PET/CT was then performed to assess the nature of the lesion and the patient's systemic condition. The MIP **(A)** showed mild radiation uptake in the T7 area (the black arrow) and increased FDG uptake in the L3 lesion area (the white arrow). In addition, there is radioactive uptake of lymph nodes in the mediastinum (the asterisk arrow), which are pathologically confirmed as inflammatory. The axial figures at the T7 level [**(B)** PET; **(C)** CT; **(D)** PET/CT fusion] showed the destruction of bone density in the vertebral body and adnexa, presenting a “fence-like” change, with a slight increase in FDG uptake and an SUVmax of 3.0 (black arrows). An axial map at the L3 level [**(E)** PET; **(F)** CT; **(G)** PET/CT fusion] showed destruction of bone density in the right portion of the vertebral body with increased FDG uptake and SUVmax of 4.5 (white arrows). Based on these imaging findings, the patient was considered to have hemangiomas. Based on pathological and immunohistochemical findings, the patient was confirmed to have plasma cell myeloma.

**Figure 3:**
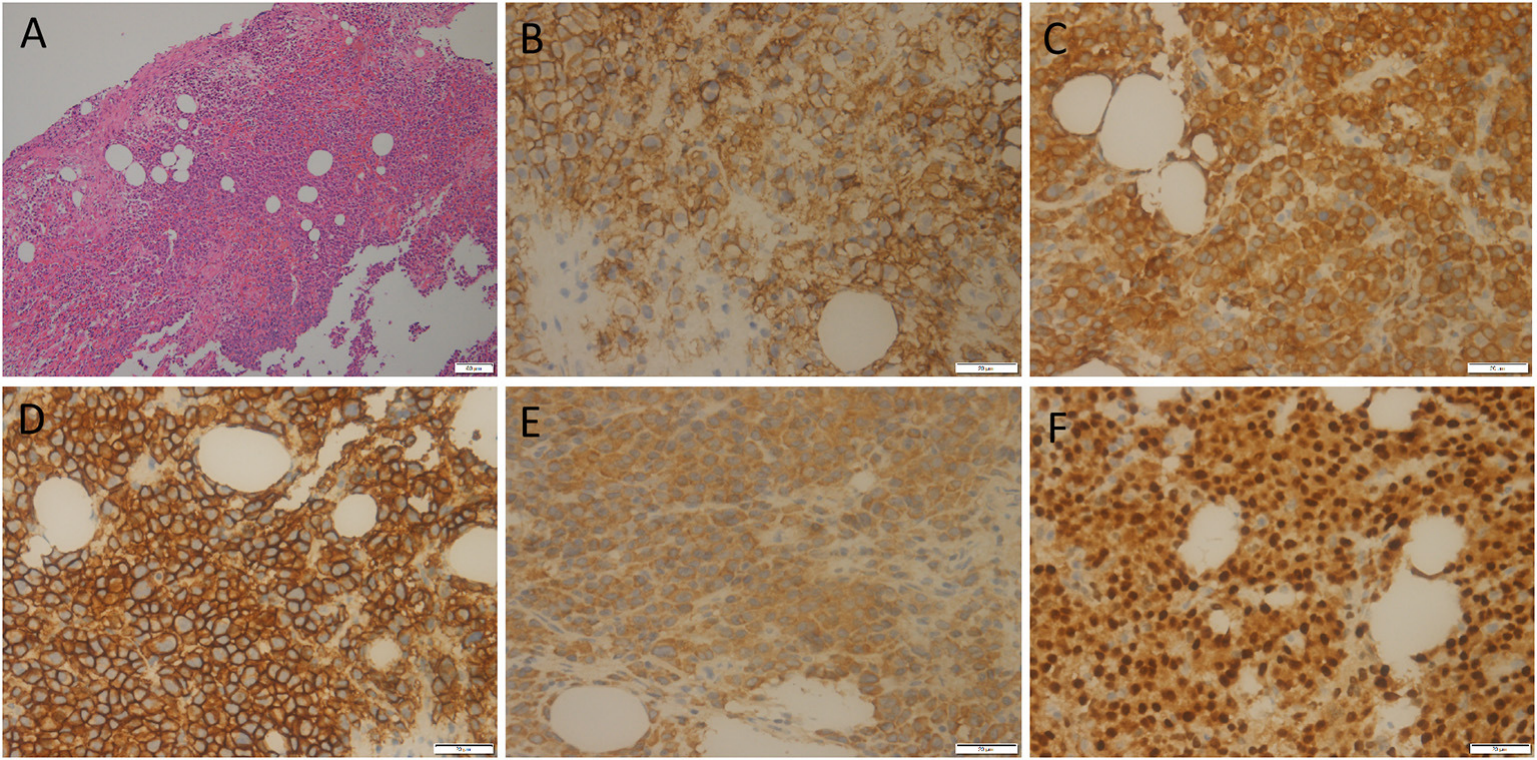
HE staining showed the diffuse distribution of round or oval plasma cells in the tumor **(A)**. Immunohistochemistry showed positive expression of tumor cells CD56 **(B)**, CD79a **(C)**, CD138 **(D)**, Kappa **(E)**, and MUM1 **(F)**.

A 55-year-old male patient presented to our hospital for more than half a year due to lower-back pain and numbness of both lower extremities. Physical examination showed that the muscle strength of both lower extremities of the patient was weakened, the lower back suffered percussion pain, and there were no positive signs in the rest of the body. The hemogram, bone marrow examination, and tumor marker values of the patient were all within the normal reference range. Imaging examinations were performed because clinicians suspected he had a lumbar disc herniation. CT showed that the patient's 7^th^ thoracic vertebral body, right vertebral arch, and 3^rd^ lumbar vertebral body had low-density bone destruction, showing “fence-like” or “honeycombing” changes (as shown in Supplementary Figure 1), which presented as uneven low T1 and high T2 signals on magnetic resonance imaging (MRI), and a contrast-enhanced scan showed marked enhancement of the lesions (as shown in Figure 1). ^18^F-FDG PET/CT showed a mild FDG concentration in the 7^th^ thoracic and 3^rd^ lumbar vertebrae, with a maximum standard uptake value (SUVmax) of 4 and 4.5, respectively, as shown in Figure 2. Based on negative laboratory results and imaging findings that simulated the “fence-like” or “honeycombing” appearance of hemangioma, the patient was considered for a diagnosis of hemangioma preoperatively. Because the diseased vertebral body has a wedge-shaped flattening and compresses the spinal cord backward, surgical treatment is necessary in order to relieve its compression symptoms. Under general anesthesia, the patient received “posterior percutaneous 3^rd^ lumbar vertebral balloon dilatation kyphoplasty, and posterior 7^th^ thoracic vertebra vertebroplasty plus posterior 7^th^ thoracic vertebral adnexectomy, spinal canal exploration and decompression, and posterior screw rod system internal fixation.” The diseased tissue removed surgically was sent for biopsy; HE staining showed the diffuse distribution of round or oval plasma cells in the tumor. Immunohistochemistry showed positive expression of tumor cells CD56, CD79a, CD138, Kappa, MUM1, CD38, Vimentin, and Lambda was weakly positive, but CD20, CD3, CK, EMA, LCA, and ALK were negative (as shown in Figure 3). Postoperatively, the patient received 10 local radiotherapy sessions over a 2-week period, each dose of approximately 30 Gy. The patient's condition was stable, and no progress was found in the follow-up for 2 years through imaging examinations. A recent X-ray examination is shown in Supplementary Figure 2.

## Discussion

Plasmacytoma is a B-cell monoclonal malignant tumor characterized by abnormal proliferation of plasma cells, accounting for about 10% of hematological malignancies (1). The etiology of the disease is unclear, but a study has found that it is closely related to herpes viruses in dendritic cells cultured from patients with myeloma, as well as to long-term exposure to industrial and agricultural toxins, ionizing radiation, chronic infection, advanced age, male sex, obesity, and family history of hematologic malignancies (2). Studies have shown that the occurrence of PCM is related to cytogenetic abnormalities, including 1q21 gene amplification, 17p deletion, and IgH rearrangement, and (t 4; 14), (t 14; 16), (t 14; 20) are considered high-risk factors (3–5). PCM is more common in the elderly, with a median age of 68 years at diagnosis, and only 35% of patients younger than 65 years at diagnosis (6). PCM often pervades the whole body bones, especially the spinal column, ribs, skull, pelvis, and other axial bones and flat bones, which is mainly manifested as progressive lytic bone destruction, which can lead to pathological fracture with the progression of the disease, and patients often come to the hospital for medical help because of bone pain (7, 8). Moreover, PCM can cause abnormal hematopoietic function, anemia, hypercalcemia, abnormal renal function, and repeated infections may appear (9). The clinical diagnosis of PCM mainly includes serological examination, bone marrow puncture, and biopsy. The proportion of monoclonal plasma cells in bone marrow ≥ 10% or clear biopsy evidence indicating bone or soft tissue plasma cell tumor (i.e., extramedullary plasma cell tumor) is the necessary condition for the diagnosis of MM, and only 3–5% of MM patients with bone marrow plasma cell proportion <10% (10). The results of the bone marrow examination in our case were all within the normal reference range, which is relatively rare.

Imaging examinations are of great significance to the localization of PCM, the extent of involvement, and the formulation of treatment plans, especially CT, MRI, and PET/CT. On CT, PCM mainly manifests as punch-like, moth-eaten, or granular low-density bone destruction areas, and some patients only show osteoporosis-like changes (11). Vertebral PCMs have different MRI appearances at different ages due to changes in the ratio of red and yellow bone marrow components. On T1WI, the area of bone destruction or bone marrow infiltration is a clearly demarcated low signal against the high signal of bone marrow fat, and contrast-enhanced T1WI scans showed mild to moderate inhomogeneous enhancement; on T2WI, the lack of contrast with a bone marrow fat signal often leads to unclear lesions; on T2WI, fat suppression due to the signal of fat was suppressed, and the high signal of the lesion was clearly displayed (12). MRI diffusion-weighted imaging (DWI) is a non-invasive method that can reflect the diffusion of water molecules in living tissues, which has a high sensitivity and specificity for the detection of bone marrow lesions in patients with PCM, and, by measuring, apparent diffusion coefficient (ADC) value can indirectly and quantitatively evaluate the damage degree of bone marrow lesions (13). Due to the infiltration and accumulation of malignant plasma cells to replace normal bone marrow tissue, the extracellular space is reduced, and the movement of water molecules is blocked, resulting in a decrease in the ADC value of PCM lesions so as to differentiate PCM from other benign lesions such as hemangioma (14). The metabolism of PCM tissue is relatively strong, and the glycolysis of normal tissue cells is significantly lower than that of the lesion. Therefore, the lesion tissue will take up a large amount of ^18^F-FDG, which shows a large amount of radioactive concentration during PET/CT imaging (15). Compared with CT and MRI, PET/CT can better display the lesion volume and metabolic activity of patients with PCM, and has higher sensitivity and specificity in the detection of extramedullary infiltration of PCM (16–18). Moreover, PET/CT also plays an important role in the efficacy evaluation after treatment and prognosis of patients with PCM, and studies have suggested that the metabolic activity of lesions, the volume of high metabolic lesions, and extramedullary infiltration are all related factors affecting the survival (19–21). In addition to FDG, ^68^ga-pentixafor, ^11^C-choline, and ^11^C-methionine were also used as PET tracers in plasma cell myeloma detection studies and showed a higher detection rate than FDG (22–24).

Spinal PCM should be differentiated from metastases, vertebral tuberculosis, hemangioma, etc. Metastatic bone tumors are more common in middle-aged and elderly people, and usually have primary tumors, which are mostly osteolytic destruction of the posterior vertebral body, with unclear boundaries and irregular shapes, often involving the vertebral arch and forming paravertebral soft tissue masses (25). Spinal tuberculosis is mostly irregular osteolytic destruction, the intervertebral space between the diseased vertebral bodies narrows and disappears, the vertebral bodies are embedded in each other, and paravertebral cold abscesses and bone bridge formation may also appear. On MRI, the diseased vertebral body usually shows an inhomogeneous low signal on T1WI and a high signal on T2WI, and the paravertebral abscess shows annular enhancement on an enhanced scan (26). The typical CT manifestations of vertebral hemangioma are decreased bone density of the affected vertebral body, with mesh or honeycomb changes, and slight swelling and thinning of the bone cortex. Two-dimensional reconstruction shows “fence-like” or “honeycombing” of the affected vertebral body, and the lesions may invade half or the entire vertebral body or accessories, with occasional paravertebral or intraspinal soft tissue masses, and non-contrast-enhanced scans; the tumor was significantly enhanced due to the presence of more vascular components (27, 28). Our patient showed “fence-like” low-density bone destruction on CT, with obvious homogeneous enhancement on enhanced MRI, and mild radioactive uptake on PET/CT, which overlapped with the imaging features of hemangioma and was misdiagnosed, which overlapped with the imaging features of hemangioma and was misdiagnosed.

The main treatment of PCM includes anti-myeloma drugs, bone-targeted drug therapy, local radiotherapy, and chemotherapy, while surgery is usually not recommended for patients with a definite diagnosis of PCM because the optimal timing of chemoradiation may be missed (29). However, when the tumor destroys the bone and causes a fracture, surgery should be performed as soon as possible when the patient has spinal cord compression with neurological symptoms, as surgery can relieve the tumor's compression on the spinal cord and avoid further aggravation of spinal cord injury caused by compression (30). At the same time, the development of individualized diagnosis and treatment plans for PCM patients with different conditions is helpful for the improvement of the patient's condition. The prognosis of patients with PCM is poor, and the literature reports that the 2- and 3-year survival rates of PCM patients with spinal cord involvement are 58 and 50%, respectively (31). Our patient has been followed up for 2 years without disease progression.

In conclusion, PCM of a single thoracic and lumbar spine is rare, and its imaging manifestations can be simulated as vertebral hemangioma, which should be used as one of its differential diagnoses. Individualized treatment plans are feasible for patients with vertebral PCM, especially those with spinal cord compression. Local radiotherapy after surgical resection of the lesion may improve the prognosis of single thoracic and lumbar PCM.

## Data availability statement

The original contributions presented in the study are included in the article/Supplementary material, further inquiries can be directed to the corresponding authors.

## Ethics statement

Written informed consent was obtained from the individual(s) for the publication of any potentially identifiable images or data included in this article.

## Author contributions

JC and PW: funding acquisition. DL: investigation. SL: methodology. XH and WX: writing–original draft. XH, XL, and JC: writing–review and editing. All authors contributed to the article and approved the submitted version.

## Funding

This study was funded by the National Natural Science Foundation of the People's Republic of China, NSFC (Grant No. 81571712), Zunyi Medical College Research Start Fund 2018ZYFY03, and QianKeHe platform talents [2017] (Grant No. 5733-035).

## Conflict of interest

The authors declare that the research was conducted in the absence of any commercial or financial relationships that could be construed as a potential conflict of interest.

## Publisher's note

All claims expressed in this article are solely those of the authors and do not necessarily represent those of their affiliated organizations, or those of the publisher, the editors and the reviewers. Any product that may be evaluated in this article, or claim that may be made by its manufacturer, is not guaranteed or endorsed by the publisher.
