# Supplementary material for: Case report: Thoracic and lumbar plasma cell myeloma mimicking hemangiomas on MRI and 18F-FDG PET/CT
Source: Front Med (Lausanne). 2022 Aug 4;9:967531. doi: 10.3389/fmed.2022.967531 (PMC9386064; doi:10.3389/fmed.2022.967531)
Supplement: Supplementary file 1 [file Data_Sheet_1.doc]

**
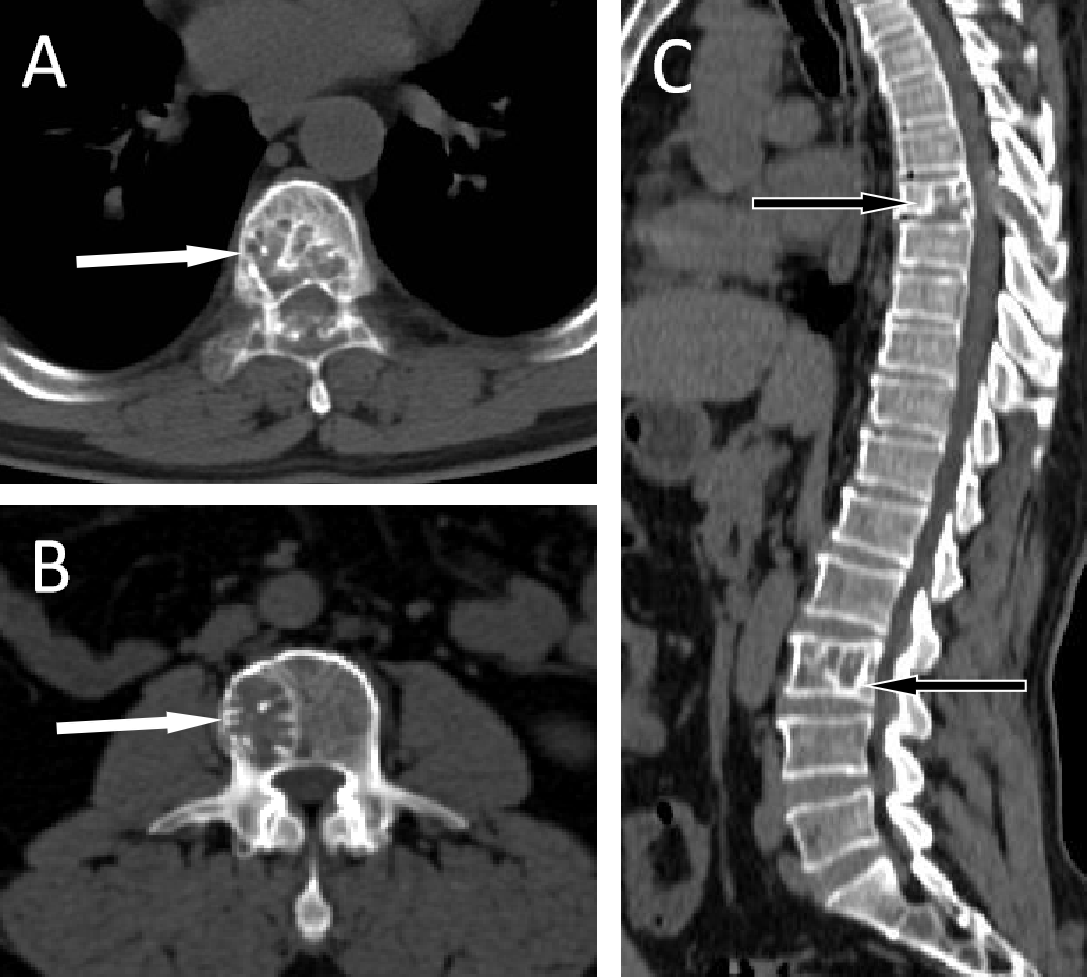
**

**Supplementary Figure 1.** Preoperative CT images of the patient: “fence-like” or “honeycomboing” hypodensity destruction of the 7th thoracic vertebrae (A, white arrow) and 3rd lumbar vertebrae (B, white arrow) ; Sagittal plane shows hypodense bone destruction with hyperdense sclerotic zone (C, black arrows).

**
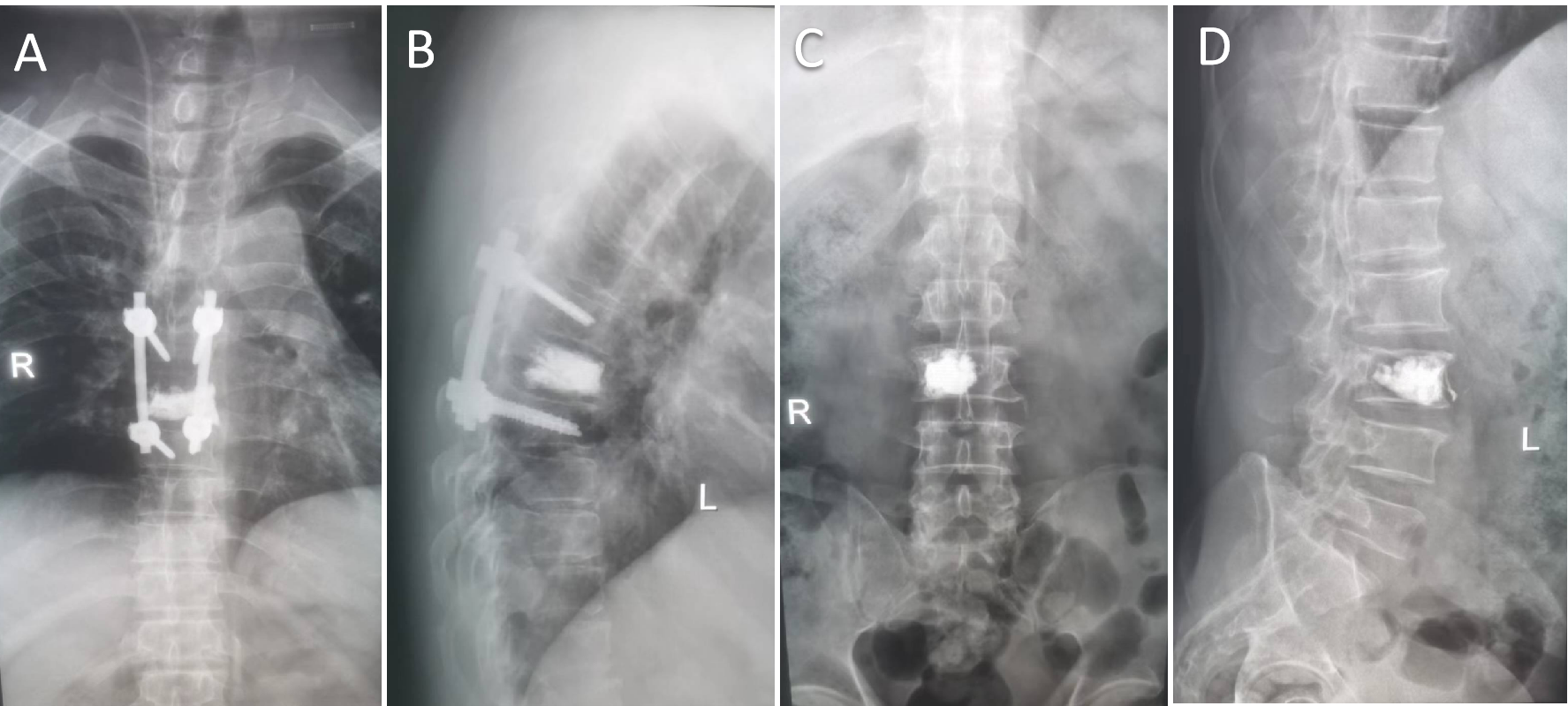
**

**Supplementary Figure 2.** X-rays of the patient 2 years after surgery: The seventh thoracic vertebra and the third lumbar vertebra showed postoperative changes, and the remaining vertebral bodies showed no obvious signs of bone destruction. (A) anterior and posterior view of thoracic spine; (B) Lateral X-ray of Thoracic Spine; (C) anterior and posterior view of the lumbar spine; (D) Lateral X-ray of Lumbar Spine.
